# Effect of Intercritical Annealing Time on Microstructure Evolution and Mechanical Properties of Low Carbon Medium Manganese Steel Subjected to Multi-Step Heat Treatment Process

**DOI:** 10.3390/ma15072425

**Published:** 2022-03-25

**Authors:** Feilong Wang, Xiaoyu Ye, Shoubin Ren, Kaihua Zhang, Xiaokai Liang, Gang Liu

**Affiliations:** 1 State Key Laboratory of New Metal Materials, University of Science and Technology Beijing, Beijing 100083, China; wfeilong@126.com; 2 State Key Laboratory of Vanadium and Titanium Resources Comprehensive Utilization, Pangang Group Research Institute Co., Ltd., Panzhihua 617000, China; yexiaoyupzh@163.com (X.Y.); zhangkaihua@163.com (K.Z.); 3 School of Metallurgical Engineering, Anhui University of Technology, Maanshan 243002, China; 4 Xichang Steel & Vanadium Co., Ltd. Pangang Group, Xichang 615000, China; renshoubin@163.com; 5 Department of Structural Steels, Central Iron and Steel Research Institute, Beijing 100081, China; liangxk@sina.com; 6 Beijing Advanced Innovation Center for Materials Genome Engineering, University of Science and Technology Beijing, Beijing 100083, China

**Keywords:** multi-step heat treatment process, intercritical annealing time, reversed austenite, microstructure, mechanical properties, medium manganese steel

## Abstract

A novel multi-step heat treatment process was performed for 0.2C–5Mn steel, and the effect of intercritical annealing (IA) durations on the microstructure evolution and mechanical properties was studied. The results showed that the content of primary reversed austenite (PRA) hardly changed as the IA time increased from 6 h to 50 h, but only less than 10% of PRA remained after being tempered at 200 °C due to the appearance of secondary martensite (SM). The final microstructure contained SM, the primary martensite (PM), and RA, which was protected by the SM so that the transformation-induced plasticity (TRIP) effect was unlikely to occur. Meanwhile, the (Ti, V, Mo)C particle sizes were 14.27, 14.68 and 15.65 nm for the intermediate processes of IA-6 h, IA-12 h, and IA-50 h, respectively. As the IA time increased from 6 h to 50 h, both the dislocation and precipitation strengthening increment decreased. As a result, the best mechanical properties were obtained from the intermediate process of IA-12 h, with a yield strength of 1115.5 MPa, tensile strength of 1573.5 MPa, and −20 °C impact energy of 30.4 J.

## 1. Introduction

For conventional low-alloy steel with a body-centered cubic structure, there is usually a conflict between strength and ductility or toughness due to the different plastic deformation mechanisms [1,2,3]. However, this problem can be effectively solved by austenite, which significantly inhibits crack nucleation and propagation, especially at low temperatures. Wang et al. reported a tensile strength of 852 MPa, the total elongation of 73% and −196 °C impact energy of 172 J for a high-Mn austenitic steel [4]. Mn, as the dominant element used to enlarge the austenite phase region, can introduce austenite with C by a suitable heat treatment process, having a lower price than Ni [5]. Medium manganese steels with Mn contents varying from 4 to 12 wt.% possess excellent comprehensive mechanical properties, for example, the product of tensile strength and total elongation is 20~60 GP·%, all of which have been successfully applied in the automobile industry, offshore structures, bridges, and so on [6,7].

It is well noted that the RA plays an important role in such steel to match strength, toughness and plasticity [1,2,3,6,7,8]. As the dominant elements in RA, C and Mn control the level of stacking fault energy, which influences the plastic deformation mechanism [9]. For low-carbon medium manganese steel, RA usually transforms into martensite to improve strength during deformation, as well as depending on the lower stacking fault energy, which is the so-called TRIP effect [10]. The TRIP effect strongly depends on the stability of RA, which is affected by several factors: chemistry, distribution, morphology and content.

Firstly, Mn and C remarkably stabilize RA [11]. When the content of C is lower than 0.05, the undercooled austenite tends to transform into ε-martensite with a close-packed hexagonal structure, which damages the toughness but increases the work hardening ability [12]. A higher Mn content promotes the larger strain partitioning of Mn, which inhibits micro-crack formation inside the ferrite and potentially changes the plastic deformation mechanism from the TRIP effect to a twinning-induced plasticity mechanism with a higher stacking fault energy [13]. Then, the lath-like RA distributed in the lath boundary has a higher stability than block RA distributed in block, packet and prior austenite grain boundaries [3,12]. Thirdly, the RA stability decreased with the increase in RA content [14]. The proper RA stability induced the proper TRIP effect, which induced the superior comprehensive mechanical properties [15]. However, all these factors stem from the ingenious chemical composition design and specific heat treatment, e.g., preservation in the α + γ two-phase region (namely, IA process) [16].

With an increase in IA temperature, the content of RA firstly increases and then decreases as the stabilizing element of Mn and C decreases in undercooled austenite and the size tends to be larger [17]. In addition, the content of RA increases with an increase in IA time, leading to a decrease in yield strength all the time [14]. This decrease in strength is usually due to the high tempering temperature. On the one hand, the RA content increases, and on the other hand, the dislocation density decreases significantly [18]. However, the low yield strength loses its application in some specific engineering structural steels. Therefore, a novel heat treatment was adopted to guarantee both a high dislocation density and certain RA content to obtain a high strength and low-temperature toughness.

## 2. Materials and Experimental Methods

### 2.1. Materials Smelting and Rolling

The nominal chemical composition of the used Fe alloy is listed in Table 1. The Fe alloy was melted in a 50 kg high-frequency vacuum induction furnace and cast into a rectangular billet with air cooling to room temperature. Subsequently, the billet was reheated to 1200 °C homogenization for 1 h, and the initial rolling temperature was about 1150 °C. After being subjected to six passes rolling, the thickness varied from 60 mm to 20 mm, and the final rolling temperature was about 850 °C, with the air cooling to room temperature.

### 2.2. Heat Treatment Process

**Figure 1:**
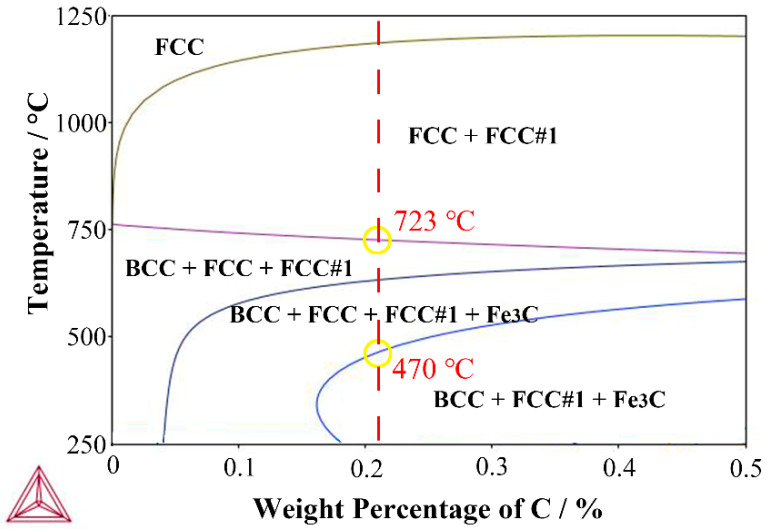
Phase diagram of the 0.2C-5Mn steel.

**Figure 2:**
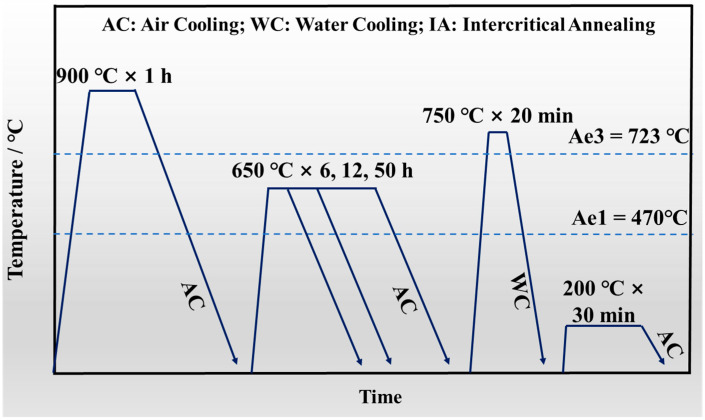
Schematic diagram of the heat treatment process.

In order to obtain RA, the Thermo-Calc software with TCFE 11.0 database was used to calculate the phase transformation temperature. The calculated Ae1 was 470 °C, while the calculated Ae3 was 723 °C in Figure 1. Given this, the rolling steel was subjected to the heat treatment process as shown in Figure 2. In order to eliminate the uneven rolling microstructure and dissolve part of the precipitation particles, the rolling steel was reheated to 900 °C for 1 h, and cooled by air, termed the normalizing process. Then, the IA process, which is in the α + γ two-phase region, was applied to form RA. Different times were used to ensure different contents of RA, and then the steels were put into a box furnace to perform the process at 750 °C for a total furnace time of 20 min, followed by water cooling to room temperature. Finally, the steels were subjected to a low-temperature tempering process (200 °C for 30 min).

### 2.3. Microstructure Characterization

The SEM specimens were machined by the electro-discharge cutting machine over different heat treatment periods. After mechanical grounding, polishing, and chemical etching in a mixed solution of 4% nitric acid alcohol solution, the specimens were observed at a FEI Quanta 650 field emission SEM for microstructure.

EBSD specimens for detecting the RA distribution and misorientation information were subjected to electrolytical polishing in a mixed solution of 10% perchloric acid and 90% absolute ethanol, and were then observed at a Zeiss Merlin SEM equipped with an OXFORD Nordlys-Nano detector. The tested area and step size were 100 μm × 75 μm and 0.15 μm, respectively. The EBSD data were post-processed by HKL Channel 5 software to obtain more detailed information.

FEI TECNAI G^2^ F20 TEM was used for observing microstructure morphology and nano-precipitation. TEM specimens were cut from each step in the heat treatment process, and were mechanically ground to near 50 μm. After being punched into several 3 mm diameter discs, the twin-jet electrolytic polisher was used to obtain a thin area for observation under a voltage of ~30 V and a temperature of −20 °C in a mixed solution of 4% perchloric acid and 96% absolute ethanol.

XRD was adopted to test the contents of RA and dislocation density of martensite. XRD samples were about 5 mm thick and were electropolished in a mixed solution of 10% chromic acid and distilled water in a current of 0.03 mA to relieve surface residual stress. The XRD line profiles were analyzed by MDI Jade 6.0 software. Diffraction peaks of (200)γ, (220)γ, (311)γ, (200)α and (211)α were considered to calculate the content of RA depending on the following equation [2,6]:

(1) $$V_{\gamma }=\frac{1.4I_{\gamma }}{I_{\alpha }+1.4I_{\gamma }}$$

where I*_α_* and I*_γ_* are the diffraction peak intensity of α-Fe and γ-Fe, respectively.

### 2.4. Secondary Phase Precipitation Characterization

The types and size distribution of precipitation phases were characterized and analyzed by carbon extraction replicas, followed by TEM observation. The specimens were from SEM samples but subjected to deeper etching in 4% nitric acid and 96% absolute ethyl alcohol about 10 min. Placed in a vacuum evaporator, the discharge at the tip of the carbon rod sprayed the carbon uniformly onto the surface of the dry samples, forming the carbon coating, about 20 nm thick [19]. Then, the sample surface with carbon film was divided into several regions in sizes of 3 mm × 3 mm by a blade. Etched in 4% nitric alcohol solution, the pieces of carbon film fell off and curled up due to low surface tension from ethyl alcohol. After further cleaning in distilled water, the dry replica samples were placed on the copper (Cu) grids. The TEM-Tecnai G^2^ F20 with nano-beam EDS was used for observing the precipitation phases. The mean precipitation particles were measured by the Image-Pro software in at least 10 areas.

### 2.5. Mechanical Property Tests

Standard round tensile specimens with the dimensions of 10 mm diameter, 50 mm gauge length, and 110 mm total length were prepared from steels subjected to the multi-step heat treatment process along the rolling direction. Tensile tests were performed at a cross-head speed of 1 mm/min at room temperature using a WE-300 hydraulic tensile testing machine equipped with the extensometer strain readings for tensile properties.

Charpy V-notch (CVN) specimens (10 mm × 10 mm × 55 mm) were machined along the rolling direction, performed on a JBN-300B impact machine at −20 °C. Considering the rise in temperature during the test, the specimens were cooled to −22 °C by liquid nitrogen. There are two samples for tensile tests, while there are three samples for −20 °C impact tests.

### 2.6. Phase Transformation Temperature Test

Cylindrical samples 10 mm long and 3 mm in diameter were prepared from the steel after being intercritically annealed at 650 °C for 50 h, and the phase transformation temperatures were estimated by a Formastor-FII thermal dilatometer. After heating to 750 °C by a linear rate of 5 °C/h and holding for 20 min to simulate the actual process, each sample was cooled to room temperature at a fixed rate of 50 °C/s. Additionally, the phase transformation temperatures were measured by the tangent method.

## 3. Results

### 3.1. Microstructure Characterization after Being Subjected to IA at 650 °C

**Figure 3:**
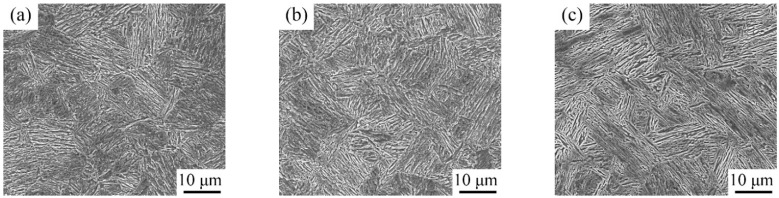
SEM images after being intercritically annealed at 650 °C: (**a**) 6 h; (**b**) 12 h; and (**c**) 50 h.

Figure 3 displays the microstructure of the 5 Mn steel after being subjected to IA at 650 °C for different periods. The microstructure was mainly lath martensite, and there were a lot of corrosion pits, which were mainly attributed to the uneven distribution of Mn [20]. Lath-like and granular morphologies were found for them. However, the martensite packet boundaries and prior austenite grain boundaries (PAGB) were hard to differentiate.

**Figure 4:**
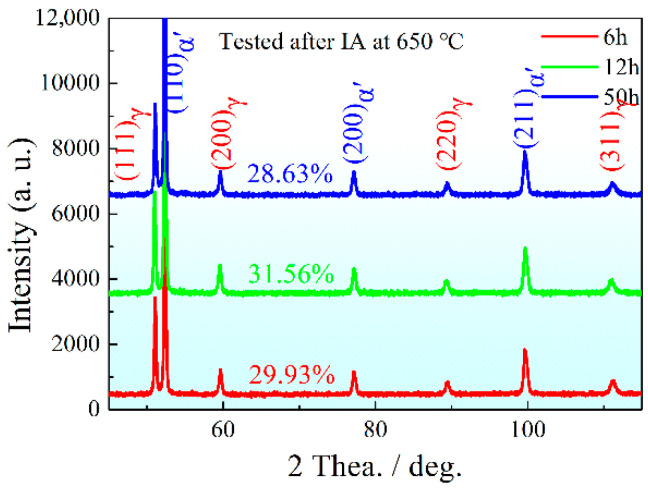
XRD line spectra after being intercritically annealed at 650 °C with different durations.

The XRD spectra of the experimental steel depict the diffraction peaks of γ-Fe and α-Fe in Figure 4. In view of the high hardenability of the 0.2C-5Mn alloying steels, very little undercooled austenite remained even if the steel was subjected to air cooling [21]. Based on Equation (1), the measured contents of PRA were 28.63%, 31.56% and 28.63%, respectively. With an increase in IA time, the contents of PRA firstly increased and then decreased, indicating that the α/γ interface migrated into the martensite lath interior [14], and the martensite transformation occurred.

**Figure 5:**
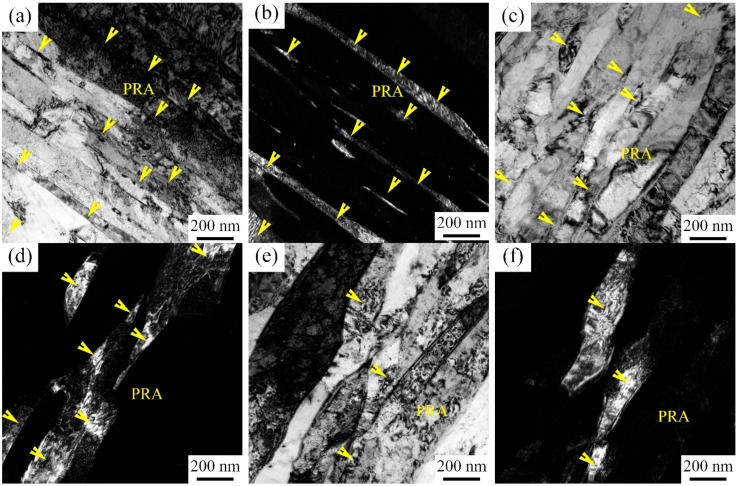
TEM images after being intercritically annealed at 650 °C: (**a**,**b**) IA-6 h; (**c**,**d**) IA-12 h; and (**e**,**f**) IA-50 h. (**a**,**c**,**e**) Bright field images; (**b**,**d**,**f**) dark field images.

The martensite lath substructure was observed by TEM as shown in Figure 5, and the lath-like PRA with some stacking faults was embedded between lath martensite. With an increase in IA time, the martensite lath coarsened from about 147 nm at 6 h to about 273 nm at 50 h. With an increase in IA time, the martensite lath was coarsened due to the incorporation of martensite lath, which is consistent with the report by Xu et al. [22]. The dislocation density did not obviously decrease based on the image contrast [23]. It can be seen from Figure 5e,f that the obvious coarsened stage occurred at the edge of martensite lath or PRA after being tempered at 650 °C for 50 h, which potentially shows the phenomenon of α/γ interface reverse migration. Moreover, a large amount of round precipitation particles with sizes from 10 nm to 30 nm were uniformly distributed in a matrix.

### 3.2. Microstructure Characterization after Being Tempered at 200 °C

**Figure 6:**
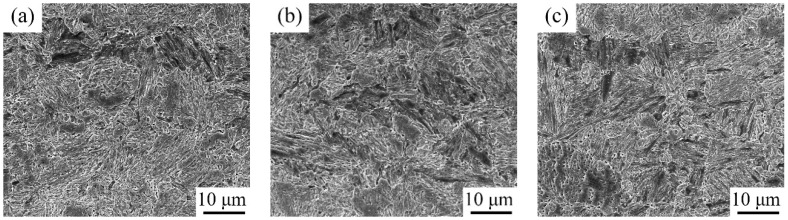
SEM images after being tempered at 200 °C: (**a**) IA-6 h; (**b**) IA-12 h; and (**c**) IA-50 h.

Different from the microstructure directly subjected to the IA process, the microstructure after being quenched at 750 °C and tempered at 200 °C lost the typical martensite lath morphology in Figure 6. There were also lots of corrosion pits along martensite substructure boundaries, showing the potential nucleation locations for PRA [12,15]. In addition, the block microstructure appeared along the clear PAGBs, which appeared after being quenched at 750 °C for 20 min. Given the short preservation and the sample homogenization time, the fraction of austenite phase transformation was limited during preserving at 750 °C. Judging from the morphology, the block microstructure was martensite with relief and some lath structures. Additionally, the block martensite size and fraction, lower than 10%, slightly increased, from IA-6 h to IA-50 h.

**Figure 7:**
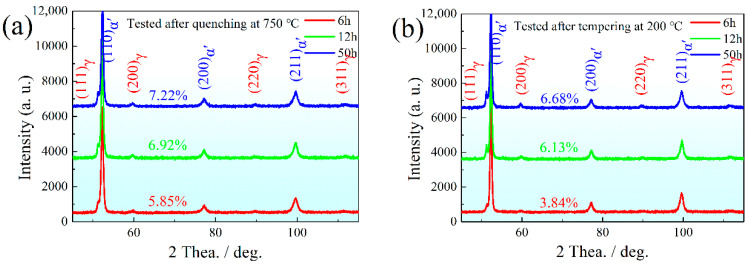
XRD line spectra: (**a**) quenching at 750 °C; (**b**) tempering at 200 °C.

The tested diffraction peak height of γ-Fe dramatically decreased compared with that after being subjected to IA process, indicating that the content of PRA decreased after being quenched at 750 °C (Figure 4 and Figure 7a). The calculated contents of RA were 7.22% at IA-6 h, 6.92% at IA-12 h and 5.85% at IA-50 h, respectively. This result suggests that more RA transformed into martensite during water cooling. After being tempered at 200 °C, the tested diffraction peak height of γ-Fe only had a slightly decrease or almost remained unchanged (Figure 7a,b), implying that the tempering process had little effect with regard to decomposing RA.

**Figure 8:**
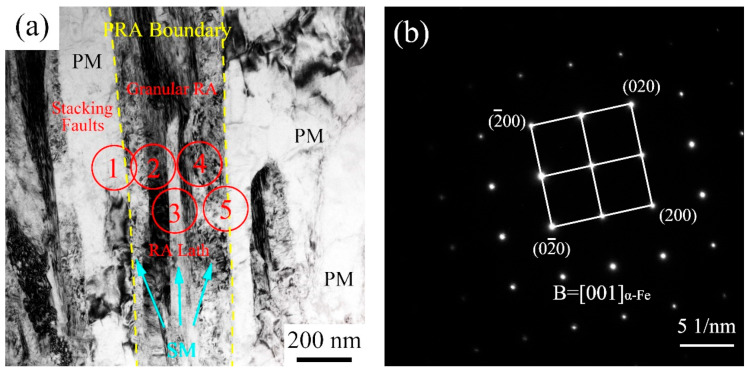

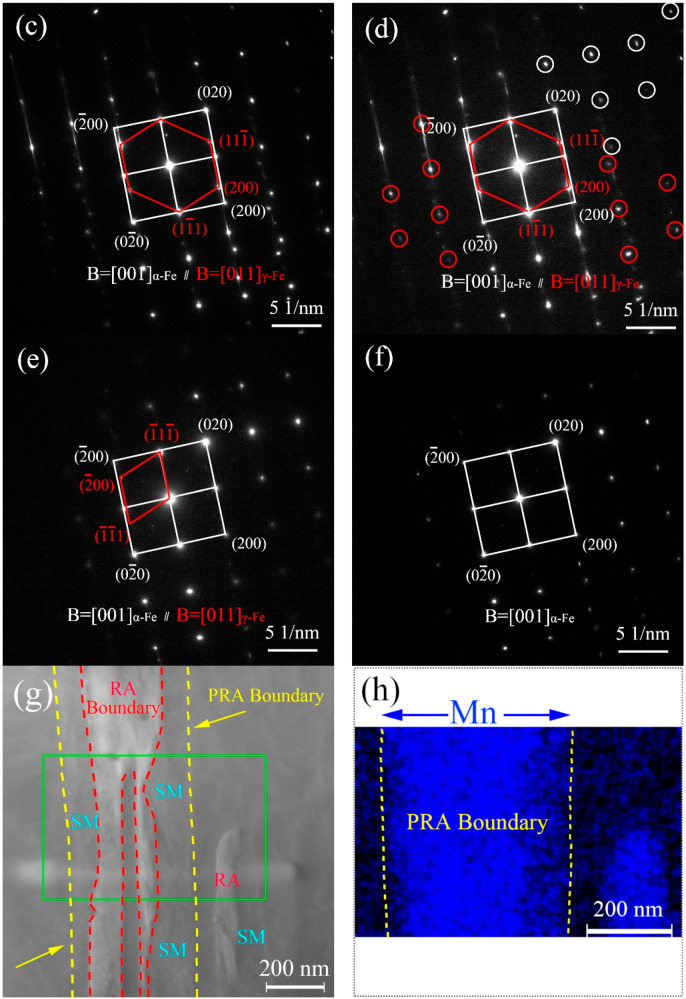
TEM images after being tempered at 200 °C with IA-12 h: (**a**) bright field image; (**b**–**f**) the SAED patterns from point 1 to point 5 marked by red circle in Figure 8a; (**g**) the image, corresponding to Figure 8a, of scanning TEM mode; (**h**) Mn map distribution.

As displayed in Figure 8a, there were complex microstructures, after being tempered at 200 °C, with the IA-12 h process with different contrasts, which could be differentiated from each other by combining the selected area electron diffraction (SAED) and TEM-EDS. Figure 8b shows the α-Fe phase along [001] zone axis of point 1 in Figure 8a. It should be noted that point 1 includes the gray area and dark gray area, but the SAED pattern shows only one set of spots of α-Fe, implying the same crystallographic orientation [15]. The gray area gives the low location dislocation density, as the potential candidate of annealed martensite. In addition, the Mn content of the dark gray area was significantly more than that of gray area. Therefore, the gray area was annealed martensite, also known as primary martensite (PM). Additionally, the dark gray area was fresh martensite, marked as SM, which was transformed from PRA during water cooling from 750 °C.

As the SAED position moved from point 1 to point 2 in Figure 8a, the diffraction spot of γ-Fe with lath morphology appeared along the zone axis of [011]. The SAED shows that there was typical Nishiyama–Wassermann (N-W) orientation relationship (OR) between SM and RA: ${\left(1\bar{1}1\right)}_{\alpha }∥{\left(1\bar{1}0\right)}_{\gamma }$ and ${\left[001\right]}_{\alpha }∥{\left[011\right]}_{\gamma }$ [24]. Moreover, the diffraction spots were elongated due to the presence of stacking faults [25]. The Mn content of was almost the same about that in dark gray area (SM), implying that SM and RA jointly belonged to PRA.

The SAED pattern at point 3 displays a similar result to that at point 2, including two phases, elongated spots, and N-W OR. However, it also shows that there were γ-Fe diffraction spots at the left and right sides, which corresponded to the dark areas in Figure 8a. Although it was the gray lath area at the center of red circle, this was an Mn-rich area in Figure 8h, showing that α-Fe represents SM. As point 3 moved to point 4, the SAED also showed the N-W OR, but γ-Fe diffraction spots were found on the left side of α-Fe, potentially revealing that the RA presented at the same position. A further right movement to point 5, the only α-Fe diffraction spots along the [001] zone axis were characterized. The Mn distribution map shows that the left was an Mn-rich area, and the right area was a low Mn area, indicating that the left side is SM and the right side is PM. In a word, the microstructure displayed SM, RA, SM, RA, and SM constituents from the left to the right side.

### 3.3. Precipitation Phase Evolution with Partition Time

**Figure 9:**
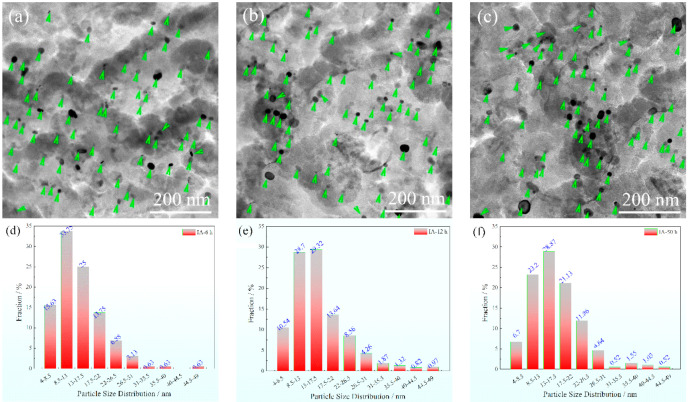
Precipitation particles’ characterization: (**a**–**c**) TEM images of precipitation particles for IA-6 h, IA-12 h and IA-50 h, respectively; (**d**–**f**) particle size distribution lower than 50 nm for IA-6 h, IA-12 h and IA-50 h, respectively.

TEM images of carbon extraction replicas are exhibited in Figure 9. A lot of nearly round particles were observed and dispersedly distributed on the matrix. The color of these nano-precipitation particles varied from low-contrast gray to black, which was due to the different crystallographic orientations. The zone axis of low-contrast gray particles was far away from the electron beam direction, while the black particles had a similar direction to the electron beam direction [15].

The statistical results for particle size distribution are shown in Figure 9d–f, and only particles below 50 nm were measured. Most of the particles were concentrated in the range 4–31 nm, especially between 8.5 and 17.5 nm. The mean particle size was 14.27, 14.68 and 15.65 nm for IA-6 h, IA-12 h, and IA-50 h, respectively, showing the coarsening process with an increase in IA time. The tested chemistry of the particles contained C, V, Mo and Ti for IA-6 h, and the fractions of V and Ti both decreased for IA-50 h. Therefore, these particles were the (Ti, V, Mo)C particles, and coarsened with the increasing fraction of Mo, which is in agreement with the report of [26].

### 3.4. Mechanical Property Tests after Being Tempered at 200 °C

The tensile properties of the tested steels are shown in Table 2. The best mechanical properties were: a tensile strength of 1573.5 MPa, yield strength of 1115.5 MPa, total elongation of 13.5%, and reduction in area of 37.0%. There were obtained by the process of normalizing at 900 °C, IA at 650 °C for 12 h, quenching at 750 °C, and tempering at 200 °C. Compared to IA-12 h, the tensile strength decreased by 55 MPa for IA-6 h and 60 MPa for IA-50 h; the yield strength decreased by 87.5 MPa for IA-6 h and 68.5 MPa for IA-50 h, respectively. The plasticity for the IA-6 h process was worse than that of IA-12 h and IA-50 h, and the latter two had almost the same plasticity.

**Figure 10:**
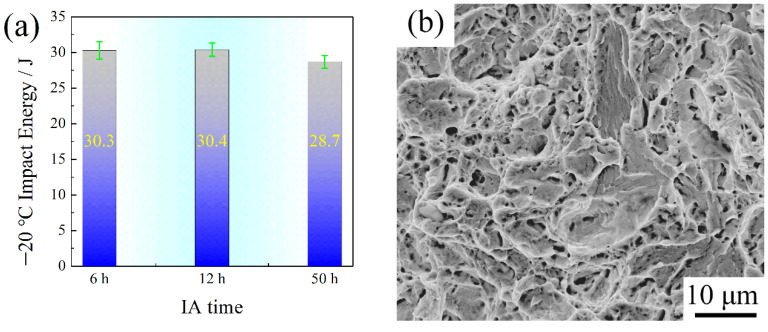

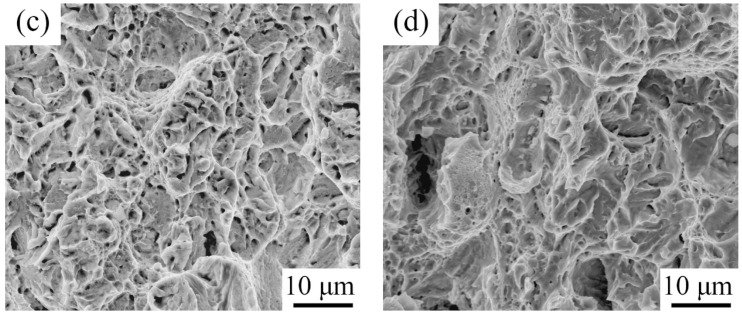
−20 °C impact energy after being tempered at 200 °C: (**a**) impact of varying energy with IA time; (**b**–**d**) are the fracture morphologies of IA-6 h, IA-12 h, and 50 h, respectively.

As presented in Figure 10a, the −20 °C impact energy decreased with an increase in IA time—30.3 J for IA-6 h, 30.4 J for IA-12 h, and 28.7 J for IA-50 h—showing that the toughness and plasticity were not completely related in mechanism. Consistent with the tensile properties, the steel subjected to an intermediate process of IA-12 h had the best toughness. They all had quasi cleavage fracture morphology, and the secondary crack mainly appeared in IA-50 h, as shown in Figure 10b–d.

## 4. Discussion

### 4.1. Microstructure Evolution Varying with IA Time

The PRA is uniformly formed during IA at 650 °C along the martensite substructure boundaries and PAGBs. Under thermodynamic conditions, the martensite is gradually replaced by austenite with an increase in temperature [14]. However, the isothermal time is also a necessary factor for kinetic conditions, which can be described by the classical Kolmogorov–Johnson–Mehl–Avrami (KJMA) theory. Additionally, the isothermal transformed fraction of PRA is expressed as [17]:

(2) $$f^{\gamma }\left(t\right)\;=f_{\mathrm{Eq}}^{\gamma }[1-\exp\left(-kt^{n}\right)]$$

where *n* is an Avrami exponent, k is a coefficient that depends on nucleation and isotropic growth rates, and $f_{\mathrm{Eq}}^{\gamma }$ is the equilibrium volume fraction of austenite. The mass fraction of austenite at equilibrium state calculated by Thermo-Calc software was 40.45% at 650 °C. Substituting the above data into Equation (2), *n* = 0.30 and k = 0.06 were obtained. Therefore, the PRA content of 36.40% was calculated at IA-50 h, while the tested content was only about 29.93%, showing that PRA partly transformed into martensite.

There were similar morphologies of PRA as the IA time increased from 6 h to 50 h, as shown in Figure 5. Hence, the size and chemical composition were the main factors to stabilize PRA. The PRA size increased from IA-6 h to IA-50 h (Figure 5a), which lost the ability to stabilize PRA [6]. In addition, the contents of C and Mn decreased as the IA time increased, as shown in Table 3, which increased the Ms temperature, implying a decreasing stability [15]:

(3) $$\mathrm{Ms}\left(K\right)\;=812\;-\;423w_{C}-30.4w_{\mathrm{Mn}}$$

where *w*_C_ and *w*_Mn_ are the element concentrations (in wt.%) of C, Mn and Al in PRA.

**Figure 11:**
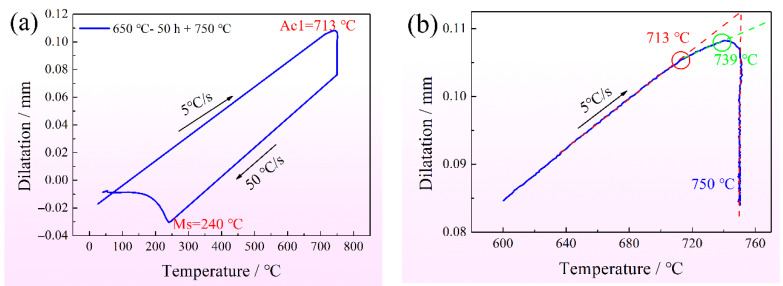
(**a**) Dilatometric curves after being intercritically annealed at 650 °C for 50 h; (**b**) magnified area near the phase transformation points.

After being subjected to the IA process, the structure mainly included two parts: one part is martensite, containing the PM and a small amount of SM, and the other part is PRA. During preservation at 750 °C, there was an inflection point deviating the linear heating stage, demonstrating that martensite partly transformed into austenite (Figure 11). It can be inferred that only the SM with high C and Mn transformed into austenite, because the PM after IA-50 h had higher contents of C and Mn compared to IA-6 h and IA-12 h, but lower than that in SM. The empirical equation of prediction Ac1 was proposed by Andrews, as follows [27]:

Ac1 = 723 − 10.7 Mn − 13.9 Ni + 29 Si + 16.9 Cr + 290 As + 6.38 W(4)

where the elements represent the mass percentage. Therefore, the SM formed at 650 °C IA for 50 h transformed into austenite, preserved at 750 °C.

There were two special phase transformation points, 713 °C and 739 °C, respectively, potentially showing the faster phase transformation from SM, and the slower phase transformation from an uneven matrix (Figure 11). After being quenched at 750 °C, PRA did not transform into martensite during water cooling (Figure 7a). Additionally, the Ms temperature of PRA of about 240 °C appeared, while the Mf temperature did not appear, showing the martensite transformation was not incomplete (Figure 11) [28]. TEM also observed the same phenomenon that PRA partly transformed into SM at the two sides and center (Figure 7). For SM formed at two sides, it was found that the content of Mn was lower than the mean level. Furthermore, the formation of SM at the PRA lath edges resulted in the expansion of volume, as displayed in Figure 11, imposing compressive stress on PRA. As the SM content increased, the maximum compressive stress was placed at the center of PRA, leading to further martensite transformation. Before and after tempering, there were similar contents of RA, showing that the tempering process has little effect with regard to decomposing the RA (Figure 8). Finally, the complex microstructure contained PM, SM, and RA, where the RA was protected by martensite.

### 4.2. Mechanical Property Evolution Varying with IA Time

The variation in mechanical properties is not significant, as shown in Table 2 and Figure 10. The most excellent mechanical properties were obtained at IA-12 h: a yield strength of 1115.5 MPa, tensile strength of 1573.5 MPa, and −20 °C impact energy of 30.4 J. Additionally, the main dislocation strengthening and precipitation strengthening mechanism were considered.

The dislocation density varying with IA time was calculated by the broadening diffraction peak [29]:

(5) $$\Delta K≅\frac{0.9}{D}+\mathrm{Nb}\sqrt{\rho }K$$

where Δ*K* is the peak width; N is a constant (0.263); D is the effective grain size (EGS); *K* is the magnitude of the diffraction vector; *K* = 2 sinθ/λ, θ and λ are the corresponding diffraction angle and wavelength of XRD; b is the magnitude of the Burgers vector and ρ is the dislocation density, which is introduced in Equation (6) to calculate the dislocation strengthening increment [18]:

(6) $$\Delta \sigma_{d}=M\alpha Gb\rho^{\frac{1}{2}}$$

where *M* is the Taylor factor, 2.75, *α* is the scale factor related to the crystal structure, 0.166, *G* is the shear modulus, 78 GPa, and *b* is the Burgers vector modulus, 0.248 nm. According to Figure 7b, the calculated dislocation density and strengthening increment are displayed in Table 4.

The (Ti, V, Mo)C precipitation strengthening increment is evaluated by Equation (7) [26]:

(7) $$\Delta \sigma_{P}=8.995\times 10^{3}\frac{f^{\frac{1}{2}}}{d}\ln\left(2.417d\right)$$

where *d* and *f* are the particle size and volume fraction of (Ti, V, Mo)C, respectively. It is worth noting that the mass fraction of (Ti, V, Mo)C calculated by Thermo-Calc at 750 °C was adopted. The (Ti, V, Mo)C size of the statistical data of Figure 9 was used. Additionally, the final calculated precipitation strengthening increments is depicted in Table 4.

The calculated dislocation density reached about 5 × 10^15^/m^2^ after being subjected to the multi-step heat treatment process. The sums of the dislocation strengthening increment and precipitation strengthening increment are 494, 481, and 453 MPa, respectively, by adopting the method of root mean square superposition [30]. The calculated result is different from the yield strength, varying from IA-6 h to IA-50 h, which is mainly due to the reasons described in the following paragraphs.

Firstly, the annealed martensite was gradually replaced by PRA, leading to the decrease in EGS. The content of PRA increased from IA-6 h to IA-50 h, which further decreased the EGS. According to the Hall–Petch equation, the yield strength increased with the decrease in EGS [18]. Secondly, there were different amounts of (Ti, V, Mo)C particles dissolved during quenching at 750 °C. The content of annealed martensite decreased from IA-6 h to IA-50 h, leading to the decrease in fine (Ti, V, Mo)C particles in annealed martensite. More fine (Ti, V, Mo)C particles were dissolved to annealed martensite basing on the dissolution principle. More importantly, as the soft phase, the higher the content of RA, the lower the yield strength [14]. The above reasons jointly caused the yield strength to increase from IA-6 h to IA-12 h, but it decreased from IA-12 h to IA-50 h (Table 2) after being subjected to the multi-step heat treatment process.

The variation tendency with regard to uniform elongation depended more on the dislocation density than the RA. According to the calculation, the dislocation density gradually decreased as the IA time increased. The high dislocation density at IA-6 h rapidly caused the dislocation entanglement and was very hard to move, leading to the first appearance of necking. Due to the low content of RA, it was hard to differentiate whether the RA was effective. Protected by the secondary martensite, the RA would not transform into fresh martensite at the initial stage of tensile deformation. When a certain deformation degree was reached before necking, the deformation distortion of secondary martensite may partly induce the transformation from RA to fresh martensite until the necking appeared.

The variation tendency of local elongation depended more on the RA than dislocation density. After necking, the entangled dislocations were the potential crack source, which needs the RA TRIP effect to release the stress concentration. In addition, fresh martensite is also an important factor to be considered for this variation tendency of local elongation. It is known that the fresh martensite contained the highest C and Mn at IA-6 h, which was the hardest phase in which to separate the matrix, causing rapid crack propagation. Therefore, the local elongation of IA-6 h was the lowest, 4.2%, while the highest local elongation of 5.0% was obtained for IA-12 h. In addition, the microstructure constituents were attributed to the low level of −20 °C impact energy (Figure 10a). The content and stability of RA were responsible for the fracture morphology evolution (Figure 10b–d). The fracture morphology of IA-50 h was coarser than that of IA-6 h and IA-12 h, which is consistent with the SEM and TEM observations. Different from the tensile deformation mechanism, the TRIP effect from RA occurred when it was subjected to the instantaneous deformation, which absorbed some impact energy so that the toughness was improved [1,2,3]. It can be seen that the RA contents were 6.68%, 6.13%, and 3.84% for IA-6 h, IA-12 h and IA-50 h, respectively. The RA content and stability were the main factors to improve the −20 °C impact energy; however, the quenched stress is also a factor that needs to be considered. The two reasons together caused almost the same impact energy between the IA-6 h and the IA-12 h, and the lowest impact energy of IA-50 h. In addition, the secondary crack appeared due to the low content of RA, leading to the crack propagation along the boundaries between primary martensite and secondary martensite or fresh martensite.

The typical mechanical properties and microstructures compared to our work are displayed in Table 5. It can be seen that the traditional medium manganese steel has a relatively low yield strength and tensile strength, which is mainly due to the appearance of ferrite. As the soft phase, ferrite and RA would firstly deform leading to the low yield strength. As this phase has a low dislocation density, the ferrite has superior plasticity and a high work hardening rate. In our work, the unique microstructure, containing PM, SM and RA, was designed to obtain good mechanical properties—a yield strength above 1100 MPa, tensile strength above 1500 MPa, and good low-temperature toughness, which shows more potential applications for medium manganese steel.

## 5. Conclusions

After being subjected to the multi-step heat treatment process, the effect of IA durations on the microstructure evolution, (Ti, V, Mo)C precipitation behavior, and mechanical properties were studied, and the main conclusions are as follows:

(1) Although the 0.2C-5Mn steel was subjected to different durations of intercritical annealing, the mechanical properties showed little change, so the IA-6 h was preferred for real production.
(2) With an increase in IA time, the contents of PRA were 28.63% at 6 h, 31.56% at 12 h and 28.63% at 50 h, respectively, and, after being tempered at 200 °C, the contents of RA were 6.68%, 6.13% and 3.84%, respectively. Finally, the microstructure was made up of PM, more than half, less than 10% of RA, and varying degrees of SM.
(3) The mean (Ti, V, Mo)C particle size increases from about 14.27 nm to 15.65 nm as the IA time varied from 6 h to 50 h. Meanwhile, the Ostwald ripening and preservation at 750 °C jointly promoted the (Ti, V, Mo)C particle spheroidization.
(4) The dislocation strengthening and (Ti, V, Mo)C precipitation strengthening increments were 679 and 104 MPa at IA-6 h, 660 and 102 MPa at IA-12 h, and 610 and 97 MPa, respectively. Although the superposed strength decreases with an increase in IA time, the high RA content, low EGS, and the high dissolution of (Ti, V, Mo)C jointly increased the yield strength from IA-6 h to IA-12 h, but decreased later.
(5) The RA surrounded by SM had high stability and its low content caused the TRIP effect to decrease, leading to the low level of −20 °C toughness.

## Figures and Tables

**Table 1:** Nominal chemical composition of the experimental steel (wt.%).

C	Si	Mn	Mo	Ti	V	P	S	Al	N	Fe
0.216	0.31	5.24	0.26	0.026	0.15	0.004	0.003	0.024	0.004	Bal.

**Table 2:** Tensile properties at room temperature after being tempered at 200 °C.

IA Time/h	Tensile Strength/MPa	Yield Strength/MPa	Yield Ratio	Uniform Elongation/%	Local Elongation/%	Total Elongation/%	Reduction in Area/%
6	1518.5 ± 2.5	1028.0 ± 12.5	0.677	7.8 ± 0	4.2 ± 0	12.0 ± 0.00	29.0 ± 0
12	1573.5 ± 21.5	1115.5 ± 24.5	0.709	8.5 ± 0.25	5.0 ± 0.25	13.5 ± 0.50	37.0 ± 0
50	1513.5 ± 11.5	1047.0 ± 24.0	0.692	9.3 ± 0.15	4.2 ± 0.15	13.5 ± 0.25	36.0 ± 0

**Table 3:** TEM-EDS of C and Mn in PRA.

Element	IA-6 h	IA-12 h	IA-50 h
C	0.38	0.34	0.27
Mn	8.40	8.04	7.38

**Table 4:** The calculated dislocation density, dislocation strengthening increment, and precipitation strengthening increment of the steel.

Samples	*ρ*/m^2^	Δ*σ_d_*/MPa	Δ*σ_P_*/MPa	$\sqrt{{\left(\Delta \sigma_{d}\right)}^{2}+{\left(\Delta \sigma_{P}\right)}^{2}}/\mathrm{MPa}$
IA-6 h	5.644 × 10^15^	483	104	494
IA-12 h	5.348 × 10^15^	470	102	481
IA-50 h	4.760 × 10^15^	443	97	453

**Table 5:** Typical mechanical properties and microstructures compared to our work.

Yield Strength/MPa	Tensile Strength/MPa	Total Elongation/%	Impact Energy/J	Microstructure	Ref.
830~500	960 ± 30	20~45	/	AM + RA	[14]
550~675	850~1015	42~48	/	AM + RA	[31]
830~600	960 ± 30	20~45	/	AM + RA	[32]
530~630	960~1030	28~45	/	α-F + RA	[33]
550~825	1025~1100	48~58	/	α-F + δ-F + RA	[34]
1028.0~1115.5	1513.5~1573.5	12.0~13.5	30 (−20 °C)	SM + RA + PM	Our work

(Note: AM is annealed martensite, α-F is α-Ferrite, and δ-F is δ-Ferrite).
